# Salaries of Health Visitors and Sanitary Inspectors

**Published:** 1920-10-23

**Authors:** 


### Salaries of Health Visitors and Sanitary Inspectors.

Should women who are health visitors and sanitary inspectors be paid salaries similar to those paid to men who do similar work?

The Lambeth Borough Council decided not long ago that the salaries paid to the female sanitary inspectors and health visitors should be put on a post-war basis at £350 per annum, rising by six annual increments of £15 and one of £10 to a maximum of £450 per annum, this being the salary paid to male sanitary inspectors.

The Ministry of Health was duly informed of the Council's decision, in order that their sanction to the proposal for equality might be obtained, and the Ministry replied that, as the proposed salaries appeared somewhat high, they wished to know what duties were carried out by the female sanitary inspectors, and also whether the work of the ladies in question could be regarded as of equal importance with the duties of male sanitary inspectors.

The General Purposes Committee and the Public Health Committee, after consultation with the medical officer of health, have replied that the duties are those laid down in the Sanitary Officers (London) Order, 1891, and out of these duties such were selected as the Council was of opinion could be more efficiently carried out, from the point of view of administration, by female than by male sanitary inspectors when the Council, in 1901, made its first appointment of a female sanitary inspector. Since then four other female sanitary inspectors have been appointed by the Council to undertake certain special duties. These special selected duties are, the Committee believes, as important as those carried out by male inspectors. The duties of health visitors are prescribed by the Health Visitors (London) Order, 1909, and relate to the wide subject of maternity and child welfare, than which no subject is more important in the public health administration. It is generally agreed that the training, qualification, and experience required by health visitors are of a high standard—quite as high as that of the training, qualification, and experience required under the Sanitary Officers (London) Order, 1891, in respect of both male and female sanitary inspectors.

The Borough Council considers that it cannot be suggested that the duties connected with maternity and child welfare are of less importance than those connected with the supervision of factories, workshops, workplaces, shops, out-workers' homes, laundries, kitchens of restaurants, hotels, coffee-houses, and dining-rooms, or that these latter duties are less important than those connected with drain laying, plumbing, or house inspections. The Council is also of opinion that it is difficult to differentiate exactly between the duties of a female sanitary inspector and a health visitor, and in this connection it would venture to remind the Ministry of Health of the Ministry's approval of a rearrangement of name by which the Council's two female sanitary inspectors were appointed as female sanitary inspectors and health visitors—a rearrangement resulting in combined appointments which became necessary on account of the overlapping of some of the duties of female sanitary inspectors and health visitors when the Maternity and Child Welfare Act was passed and the work of maternity and child welfare extended in consequence throughout the borough.

Female sanitary inspectors have to undergo the same professional training as male sanitary inspectors, and to pass the same qualifying examinations; whilst health visitors have also to be certificated, and for that purpose have to possess qualifications of an equally high standard and to undergo equally stringent training as (if not more stringent training than) inspectors. Many health visitors qualify also as inspectors, and *vice versâ*. The Women Sanitary Inspectors' and Health Visitors' Association has affirmed the principle of equal pay for men and women officials working side by side in public health departments, and the Association claims that the duties performed by women public health officers (1) are of a highly specialised character, (2) that for their due performance a long and highly technical training is required, (3) are of an arduous and exacting nature, and (4) are of no less value to the community than are those performed by men sanitary inspectors or inspectors of nuisances. The Borough Council also had before it a letter from the General Council of the Sanitary Inspectors' Association putting forward for sanitary inspectors (without any distinction of sex) a suggested scale of post-war salaries of £350 per annum, rising to £500.

